# How Does Sustainable Leadership Affect Environmental Innovation Strategy Adoption? The Mediating Role of Environmental Identity

**DOI:** 10.3390/ijerph20010894

**Published:** 2023-01-03

**Authors:** Ling Hu, Tai-Wei Chang, Yue-Shi Lee, Show-Jane Yen, Chih-Wen Ting

**Affiliations:** 1 Department of Finance, Hsing Wu University, New Taipei City 244, Taiwan; 2 Graduate School of Resources Management and Decision Science, National Defense University, Taipei 112, Taiwan; 3 Department of Computer Science and Information Engineering, Ming Chuan University, Taoyuan 333, Taiwan; 4 Department of Finance, Nanhua University, Chiayi 622, Taiwan

**Keywords:** environmental identity, environmental innovation strategies, sustainable development, sustainable businesses, sustainable leadership

## Abstract

This paper uses social identity theory to develop an environmental identity theory and interpret why sustainable leadership can influence environmental identity, which in turn, results in environmental innovation strategy (EIS) adoption. Data were collected from 90 samples of technology manufacturing firms in Taiwan, and a latent growth curve model was employed to analyze the longitudinal data. The research results confirm all hypotheses. Mediating analysis also supports the environmental identity as a mediating role between sustainable leadership and EIS. Indeed, past studies have not explored the mechanism studied in this paper, a novel mechanism which can not only advance the literature on sustainable development but also help companies to realize sustainable development through environmental innovation strategy adoption.

## 1. Introduction

A sustainable development strategy has been confirmed as one of the key sources of sustainability [1,2,3]. Therefore, the main goal of this research is to explain why firms adopt an environmental innovation strategy (EIS) through the organizational leadership mechanism (e.g., sustainable leadership), a pathway which previous research has not addressed. EIS refers to the strategy used to implement environmental innovations for the betterment of manufacturing activities [4,5,6]. In addition, air pollution alone has caused economic losses reaching 8 billion U.S. dollars worldwide [7], so understanding the drivers of EIS is an important issue [8,9,10]. However, previous researchers exploring the antecedents of EIS adoption almost exclusively employ the perspective of institutional regulation in order to predict environmental innovation strategy adoption [1,9]. Although these studies contribute to the furthered understanding of EIS adoption, research on the antecedents of EIS adoption is still lacking [11]. This paper argues for a new category to provide an incremental contribution to the literature on EIS. That is to say, the present paper borrows from social identity theory [12] in order to propose a new concept of environment identity “—including environment categorization, environment commitment, and environment esteem”, and describes how sustainable leadership can positively influence environment identity, which consequently positively influences EIS adoption. Sustainable leadership refers to when a leader guides his or her subordinates’ behaviors toward sustainable development instead of financial performance [13]. For example, a sustainable leader will employ the values of social responsibility, environmental responsibility, and ethical responsibility to guide his/her subordinates to realize company sustainability. Indeed, corporations have been starting to advance environmental performance by implementing EIS [14], but a limited number of studies consider methods to increase EIS adoption through sustainable leadership. Therefore, the first goal of this paper is to explore why sustainable leadership influences EIS adoption.

Social identity theory suggests that an individual draws identity from the cognitive, emotional, and evaluated dimsensions from his or her group members [12]. Previous studies have employed unidimensional variable (e.g., organizational identification) to measure social identity [15,16]. This paper divides social identity into cognitive identification, evaluative identification, and emotional identification, because individuals who an individual’s identification with a work group cognitively does not necessarily identify with the work group evaluatively or with the work group emotionally. This paper argues for the novel concept of “environmental identity” based on Tajfel’s [12] cognitive identification, evaluative identification, and emotional identification. The environmental identity of the TMT is the extent to which the TMT’s self-identification toward the environment, and it can increases the firm’s adoption of EIS. Therefore, the second goal of this paper is thus to explore the mediating role of environment identity.

Finally, since past empirical studies have been severely lacking in terms of longitudinal data collection and causality, this paper focuses on the growth perspective of environmental identity and environmental innovation strategy. That said, past empirical research has included a cross-sectional survey, so it has been overlooked that individuals may moderate their attitudes or intentions based on their environmental context. Therefore, the third goal of this paper is to use a latent growth curve model [17,18,19] with longitudinal data to fill this research gap. A total of 90 samples were obtained from three time points to validate our research model.

## 2. Literature Review

**Figure 1:**
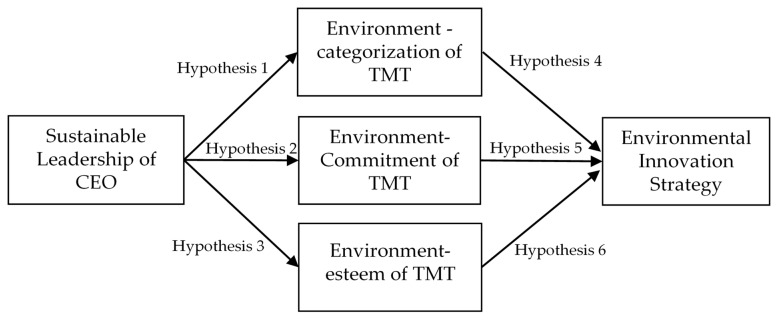
Research model of this research.

This paper proposes a novel model of environmental identity to interpret its antecedent and outcome (Figure 1).

### 2.1. Sustainable Leadership

Although leadership research in organizations has made great progress, the literature on leadership still needs to continuously emphasize leadership effectiveness within the organization [20], such as ethical leadership for leadership behaviors that achieve ethical goals [21], entrepreneurial leadership for satisfying entrepreneurship [22], and safety leadership that guides employees toward safety [23]. Subsequent research has found that leadership can be applied to the field of sustainable development to form a new concept—sustainable leadership [24,25,26].

Past research on sustainable leadership has explored its relationship with sustainable performance and added different mediating variables [24,27], but few studies have combined sustainability leadership with environmental innovation strategies through the mediating effect of the environmental identity of TMT.

### 2.2. Environmental Identity Theory

Social identity theory denotes the extent to which an individual is acquainted with identification by the cognition, emotion, and appraisal of meaning and meaningfulness of his or her group members [12]. This paper proposes that social identity theory should be classified as cognitive identification (members’ cognitive awareness within workgroups), evaluative identification (a positive evaluation of workgroups), and emotional identification (emotional attachment to workgroups). This paper employs self-categorization (a cognitive classification process of self and group) [28], group self-esteem (an individual’s positive evaluation for his or her group members) [29], and affective commitment (emotional attachment in the group) [30] as representative variables for cognitive identification, evaluative identification, and emotional identification.

This paper further proposes environmental identity, including environmental self-categorization (a cognitive classification process of self and environmental concern), environmental self-esteem (an individual’s positive evaluation of self-environmental concern), and environmental affective commitment (emotional attachment to the environmental concern).

### 2.3. Environmental Innovation Strategy

Due to climate change, pollution, poverty, etc., people’s lives are becoming more and more urgent, and sustainable business strategies are the key to changing these problems [14]. The goal of a sustainable business strategy is to have a positive impact on society and the environment, thus forming a new concept—the triple bottom line. The triple bottom line is an important concept, and it proposes that companies must pay attention to their social and environmental impacts beyond financial performance, which is beneficial to the sustainable development of profits, people, and the planet [31].

In this paper, we adopt EIS as a key sustainable business strategy, because EIS implies the strategy of implementing environmental innovations for better manufacturing activities [4,5,6]. Not only can EIS increase the profits of enterprises, but it can also reduce the pollution of the earth and form a sustainable human living environment, which meets the triple bottom line.

### 2.4. The Sustainable Leadership and the Environmental Identity of the TMT

Sustainable leadership can shape the TMT’s environmental self-categorization, environmental self-esteem, and environmental affective commitment to meet environmentally sustainable development, which confirms the relationship between sustainable leadership and the environmental identity of the TMT.

First, sustainable leadership can shape the TMT’s behaviors and values toward the sustainable development values of the organization [32], so the TMT’s self-concepts will meet environmentally sustainable development, which is similar to the concept of environmental self-categorization. We address the first hypothesis as follows:

**Hypothesis** **1:** *Sustainable leadership positively influences the environmental self-categorization of the TMT*.

Second, past research also addresses the idea that leadership can transform subordinates’ commitment toward organizational goals, visions, and missions [33,34,35], so sustainable leadership should have a similar impact in transforming a TMT’s environmental affective commitment. We address the second hypothesis as follows:

**Hypothesis** **2:** *Sustainable leadership positively influences the environmental affective commitment of the TMT.*

Finally, the process of leadership can also bring meaningful challenges to employees’ jobs and thereby increase their self-esteem [36,37], so sustainable leadership should have a similar impact in transforming a TMT’s environmental self-esteem. We address the third hypothesis as follows:

**Hypothesis** **3:** *Sustainable leadership positively influences the environmental self-esteem of the TMT.*

### 2.5. The Environmental Identity of TMT and Environmental Innovation Strategy Adoption

The environmental identity of a TMT is the extent to which the TMT’s self-value for the environment, and it can reduce self-interests to align with the environmental mission and goals, which denotes the TMT’s preference toward EIS adoption. 

First, environmental categorization denotes the cognitive classification process of self- and environmental concern. A TMT with high levels of environmental categorization should prefer EIS adoption, because the TMT believes that the self and the environment are one. We address the fourth hypothesis as follows:

**Hypothesis** **4:** *The environment-categorization of the TMT positively influences the firm’s EIS adoption.*

Second, the environmental affective commitment denotes the emotional attachment to the environmental concern. A TMT with high levels of environment commitment should prefer EIS adoption because of the TMT’s effects attached to the environmental concern. We address the fifth hypothesis as follows:

**Hypothesis** **5:** *The environment-commitment of the TMT positively influences the firm’s EIS adoption.*

Finally, environment esteem denotes an individual’s faith in a positive evaluation of the environmental concern. A TMT with high levels of environmental self-esteem should prefer EIS adoption because of the TMT’s faith in the positive evaluation of the environmental concern. We address the sixth hypothesis as follows:

**Hypothesis** **6:** *The environmental esteem of the TMT positively influences the firm’s EIS adoption*.

### 2.6. The Mediating Role of the Environmental Identity of the TMT

The environmental identity of TMT is the extent to which the TMT’s self-conceptualizes toward the environment, and a TMT who has a high level of environmental identity will affect a company’s strategic choice [38]. In addition, based on upper echelons theory [39], a TMT’s decision-making processes are caused by supervisors’ characteristics, and leadership is a key characteristic of a supervisor [40,41,42,43], supporting the mediating role of environmental identity. Indeed, as discussed above, sustainable leadership promotes the environmental identity of the TMT, and the environmental identity of the TMT will influence a company’s strategic choice. We address the fifth hypothesis as follows:

**Hypothesis** **7:** *The environmental identity mediates the relationship between sustainable leadership and a firm’s EIS adoption.*

## 3. Materials and Methods

### 3.1. Sampling 

We queried 90 technology manufacturing firms in Taiwan, and 90 members of TMTs agreed to assist with our survey. We sent 90 emails to request these TMTs to evaluate the environmental innovation strategy adoption and their sustainable leadership, environment categorization, environment commitment, and environment esteem at three time points. The respondents were all male. The respondents had a college degree or above (20% are masters). In addition, their average work experience was 10 years. We took three months to lag, because previous studies adopted a similar framework [11,44,45], which stated that three months is a timeframe over the course of which attitude changes are visible.

### 3.2. Measures

The translation quality of the Chinese questionnaire into English was confirmed through a backward translation design [46], and a seven-point Likert scale was employed to evaluate the items. The scale is shown in Appendix A.

This paper used the scale of McCann and Holt [47] to evaluate sustainable leadership. The scales of environment categorization, environment commitment, and environment esteem were developed through Ellemers et al.’s [48] self-categorization scale, Allen and Meyer’s [30] affective commitment, and Bergami and Bagozzi’s [29] self-esteem scale. Example items are “The TMT likes environmental management and protection,” “The problems of environmental management and protection are the problems of the TMT,” and “The TMT feels confident in executing environmental management and protection”. This paper used the scale of Soewarno et al. [6] to evaluate environmental innovation strategy. 

### 3.3. Validation

The descriptive and correlation analyses are shown in Table 1. An analysis process of the confirmatory factor was performed in order to test the average variance extracted, model fit, and composite reliability. These analysis results conform to Fornell and Larcker’s [49] suggestion, as shown in Table 2. 

### 3.4. Latent Growth Curve Model

As the longitudinal data should employ longitudinal statistical techniques, this paper employs the latent growth curve model. A latent growth curve model was formed in order to analyze the changes to variables and their causal relationships over time [17,18,19]. For example, this paper proposes that sustainable leadership at the first time point will induce the positive growth of c over time. In addition, positive growth of environment categorization, environment commitment, and environment esteem will also result in the positive growth of environmental innovation strategy over time.

The latent growth curve model is a modified version of the structural equation model and has been popularly used in many investigations [50,51,52]. The benefit of the latent growth curve model is to analyze variables’ growth, which can confirm a causal relationship between these variables. This paper uses data from three time points, and the latent growth curve model can test these variables in the theoretical framework (please see Figure 1).

This paper examines environment categorization, environment commitment, environment esteem, and environmental innovation strategy as state-like variables and uses the latent growth curve model to analyze these state-like variables. Indeed, the latent growth curve model has gained ubiquitous acceptance as a powerful approach for longitudinal change to address the above gaps, and it can handle the way sustainable leadership causes the positive growth of environmental innovation strategy through the mediating effect of environment categorization, environment commitment, and environment esteem.

## 4. Analysis Results

**Figure 2:**
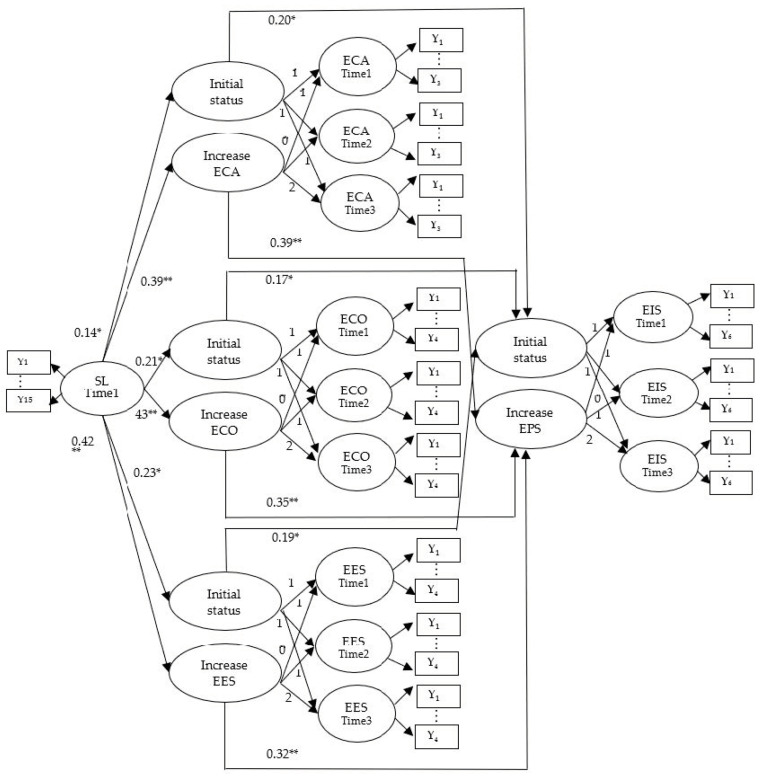
The latent growth curve model of this paper. Note: SL = sustainable leadership; ECA = environment categorization; ECO = environment commitment; EES = environment esteem; EIS = environmental innovation strategy. Yn = measurement items. * *p* < 0.05; ** *p* < 0.01. Time 2: three months after Time 1; Time 3: three months after Time 2.

This paper used the latent growth curve model to analyze the relationship between sustainable leadership, environment categorization, environmental commitment, environment esteem, and environmental innovation strategy, which was shown in Table 3 and Figure 2.

Sustainable leadership at the first time point significantly influences the growth of environment categorization (*β* = 0.39, *p* < 0.01), environment commitment (*β* = 0.43, *p* < 0.01), and environment esteem (*β* = 0.42, *p* < 0.01) over time, so Hypothesis 1, 2, and 3 are validated. That said, when TMTs perceive sustainable leadership, they develop their environment categorization, environmental commitment, and environment esteem.

Growth of environment categorization (*β* = 0.39, *p* < 0.01), environmental commitment (*β* = 0.35, *p* < 0.01), and environment esteem (*β* = 0.32, *p* < 0.01) significantly influence the growth of environmental innovation strategy adoption, so Hypotheses 4, 5, and 6 are validated. That said, the growth of environment categorization, environmental commitment, and environment esteem will lead to the growth of environmental innovation strategy adoption by technology manufacturing firms.

In order to test the mediating effects of environment categorization, environment commitment, and environment esteem, this paper adopts Baron and Kenny’s [53] procedure to analyze the relationship between sustainable leadership, environment categorization, environment commitment, and environment esteem, and EIS. First, the environment categorization (*β* = 0.39, *p* < 0.01), environment commitment (*β* = 0.43, *p* < 0.01), and environment esteem (*β* = 0.42, *p* < 0.01) were regressed on sustainable leadership, and the results showed that all coefficients were significant. Second, EIS was regressed on environment categorization (*β* = 0.39, *p* < 0.01), environment commitment (*β* = 0.35, *p* < 0.01), and environment esteem (*β* = 0.32, *p* < 0.01), and the results showed that all coefficients were significant. Finally, the environment categorization (*β* = 0.21, *p* < 0.05), environment commitment (*β* = 0.25, *p* < 0.05), environment esteem (*β* = 0.23, *p* < 0.05), and EIS (*β* = 0.15, *p* < 0.1) were regressed on sustainable leadership, and the results showed that the coefficient of EIS was not significant, which indicates that environment identity was a mediating variable. 

## 5. Discussion

### 5.1. Theoretical Implications

This paper borrows from social identity theory [12] to develop the novel environmental identity theory and explores its antecedent and outcome. Indeed, ignoring the environmental identity theory in previous studies caused a theoretical gap. Therefore, this paper provides an incremental contribution to the field of sustainable development. 

This paper proposes a linkage between sustainable leadership, environmental identity, and EIS adoption in order to open a novel mediating mechanism, which can provide an organizational management strategy (e.g., sustainable leadership) for contemporary firms to realize sustainable development through implementing EIS. Indeed, Baron and Kenny’s [53] procedure has supported the key mediating mechanisms of environment categorization, environment commitment, and environment esteem, so the environmental identity plays an important role in transforming sustainable leadership into the adoption of EIS, which opens an important black box for the relationship between sustainable leadership and EIS adoption. In addition, based on the coefficients of the environment categorization (β = 0.39, *p* < 0.01), environment commitment (β = 0.43, *p* < 0.01), and environment esteem (β = 0.42, *p* < 0.01), these effects are similar, so the three variables are equally important.

Finally, this paper is the first to link organizational management mechanisms and strategic choice mechanisms, so this paper has a cross-field contribution to the literature on leadership and the sustainability.

### 5.2. Practical Implications

In order to achieve sustainable development, this paper proposes a possible pathway to implement EIS through sustainable leadership, and the environmental identity of the TMT is a key mediating role of EIS, which is similar to past perspectives [54]. Besides encouraging companies to adopt EIS, another feasible approach is to establish a good green working atmosphere to increase environmental identity.

Finally, to implement sustainable leadership, human resources executives should consider including sustainable leadership in annual education and training, because sustainable leadership is the key driver of EIS adoption.

### 5.3. Limitations

This paper borrows from social identity theory [12] in order to address the novel EIS adoption model but generates no empirical data to verify its statistical reliability and validity, leaving further research to test it.

This paper employs sustainable leadership as a key driver to promote the environment identity theory, but other leadership styles may have a similar effect. Future research needs to test whether different leadership styles are effective for environmental identity theory in different contexts. Previous research has proven that ethical leadership has a positive effect on environment-related variables [55]. 

Finally, in addition to the environmental identity theory, there may be other important variables that influence environmental sustainability. For example, sustainable consumption may be another possible pathway to achieve environmental sustainability [56,57]. Future research should explore other key variables to complement the EIS adoption model.

## 6. Conclusions

This paper sets up a milestone in realizing sustainable development of technology manufacturing firms by the linking of sustainable leadership, environmental identity, and environmental innovation strategy. In addition, this paper also examines the relationship between sustainable leadership, environmental identity, and environmental innovation strategy through the longitudinal data and latent growth curve model in order to confirm the driving variable of environmental innovation strategy and to fill the gap in the exploration of the driving strategy behind environmental innovation strategy. That said, technology manufacturing companies can adopt sustainable leadership as a management strategy in order to enable companies to implement environmental innovation strategies. In addition, this paper uses the latent growth curve model to detect dynamic changes in environmental identity and environmental innovation strategy, in order to fill the other gap in state-like variables, and can enrich sustainable literature through the latent growth curve model.

## Figures and Tables

**Table 1:** Descriptive and correlations analysis.

Variables	Mean	Standard Deviation	SLE	ECA	ECO	EES	EIS
SLE	5.31	0.92	1				
ECA	5.24	0.86	0.41	1			
ECO	5.12	0.83	0.48	0.23	1		
EES	5.13	0.81	0.47	0.25	0.27	1	
EIS	4.99	0.93	0.27	0.41	0.39	0.37	1

Notes: SLE = sustainable leadership, ECA = environment categorization, ECO = environment commitment, EES = environment esteem, EIS = environmental innovation strategy.

**Table 2:** The analysis technique of confirmatory factor.

Constructs	Items	λ	Cronbach’s α	Composite Reliability	Average Variation Extracted
Sustainable Leadership	SLE01	0.701 **	0.937	0.89	0.61
	SLE02	0.765 **			
	SLE03	0.676 ***			
	SLE04	0.895 **			
	SLE05	0.748 **			
	SLE06	0.775 **			
	SLE07	0.778 **			
	SLE08	0.785 **			
	SLE09	0.718 **			
	SLE10	0.815 **			
	SLE11	0.788 **			
	SLE12	0.725 **			
	SLE13	0.798 **			
	SLE14	0.725 **			
	SLE15	0.758 **			
	SLE16	0.725 **			
Environment-categorization	ECA01	0.810 **	0.893	0.84	0.65
	ECA02	0.822 **			
	ECA03	0.790 **			
Environment-commitment	ECO01	0.830 **	0.913	0.88	0.65
	ECO02	0.832 **			
	ECO03	0.780 **			
	ECO03	0.790 **			
Environment-esteem	EES01	0.789 **	0.869	0.87	0.63
	EES02	0.811 **			
	EES03	0.808 **			
	EES04	0.759 **			
Environmental Innovation Strategy	EIS01	0.799 **	0.921	0.89	0.60
	EIS02	0.802 **			
	EIS03	0.758 **			
	EIS04	0.799 **			
	EIS05	0.718 **			
	EIS06	0.739 **			

Notes: (1) **: *p* < 0.01, ***: *p* < 0.001; (2) RMR = 0.048; RMSEA = 0.042; GFI = 0.92; CFI = 0.91; NFI = 0.93.

**Table 3:** The analysis results of the latent growth model.

Hypothesis	Pathway	Coefficient
H_1_	SL → ECA	0.39 **
H_2_	SL → EES	0.43 **
H_3_	SL → ECO	0.42 **
H_4_	ECA → EIS	0.39 **
H_5_	EES → EIS	0.35 **
H_6_	ECO → EIS	0.32 **

Notes: SL = sustainable leadership; ECA = environment categorization; ECO = environment commitment; EES = environment esteem; EIS = environmental innovation strategy. **: *p* < 0.01.

## Appendix A

**Table A1:** The evaluation items.

Variable	Evaluation Items
Sustainable Leadership	1. My leader acts in a sustainable, socially responsible manner. 2. My leader acts in a sustainable, environmentally responsible manner. 3. My leader acts in a sustainable, ethically responsible manner. 4. My leader’s decisions are made while considering the entire organization. 5. My leader’s management officially recognizes when a mistake is made that affects sustainability. 6. My leader is willing to correct mistakes that affect sustainability. 7. My leader attempts to use unique innovative methods to resolve sustainability issues. 8. My leader attempts to create wealth through sustainable efforts. 9. My leader puts purpose before profit. 10. My leader balances sustainable social responsibility with profits. 11. My leader demonstrates sustainability by persevering through all types of change. 12. My leader is concerned about how sustainability affects employees. 13. My leader communicates sustainability decisions to all involved. 14. My leader attempts to build a culture of sustainability through communication efforts. 15. My leader has a plan to demonstrate sustainability when hiring, promoting employees, and replacing leaders.
Environmental self-categorization	1. Our team identifies … 2. Our team likes the … 3. Our team is an import reflection of the …
Environmental affective commitment	1. Our team will very much enjoy executing the … 2. The problems of … are problems of ours. 3. Our team is emotionally attached to … 4. Our team has strong sense of belonging to …
Environmental self-esteem.	1. Our team feels confident executing the … 2. Employees respect and admire our team for executing the … 3. Our team is smart to execute the … 4. Our team is confident of understanding the …
Environmental Innovation Strategy	1. My company will choose the least-polluting material for product development or design. 2. My company will design products using the least amount of materials. 3. My company will design products that are easy to recycle, reuse and decompose. 4. My company’s manufacturing process reduces the consumption of water, electricity, coal or oil consumption. 5. My company has effectively reduced the discharge of harmful substances or waste during the product manufacturing process. 6. My company reduces the use of materials in the product manufacturing process.
